# Supplementary material for: Tumor Genomic and Transcriptomic Analysis Integrated With Liquid Biopsy ctDNA Monitoring: Analytical Validation and Clinical Insights
Source: Cancer Med. 2025 Dec 8;14(23):e71465. doi: 10.1002/cam4.71465 (PMC12685469; doi:10.1002/cam4.71465)
Supplement: Supplementary file 1 — Table S1: List of clinical samples and sequencing data used in this study. Table S2: List of 504 genes. Table S3: List of reference samples used in this study. Figure S1: Performance of FFPE DNA sequencing using high‐density probes to determine copy number variation. (A) Frequency of homozygous MTAP‐CDKN2A deletion in lung cancer samples having other actionable mutations (n = 39). (B) In silico simulation of different VAFs for detection of large genomic rearrangement (LGR) in BRCA1/2 genes. (C) Percentage of mutated BRCA1/2 (mBRCA) and wild‐type BRCA1/2 (wtBRCA) in ovarian cancer samples (n = 169). Figure S2: Performance of FFPE DNA and mRNA sequencing to detect fusion. (A) In fusion‐positive clinical samples, mRNA profiling captured more fusion events and had broader coverage of fusion genes and partners than DNA profiling. (B) mRNA profiling could detect MET Ex14 skipping when no DNA mutation was identified in lung cancer samples. Figure S3: Performance of mRNA sequencing to predict cancer tissue of origin. (A) Two‐ dimensional Uniform Manifold Approximation and Projection for Dimension Reduction (UMAP) using 3 gene sets applied in 32 cancer types in the training dataset revealed distinct clusters corresponding to different cancer types (n = 2803). (B) Sensitivity and specificity of optimized ensemble models in the testing dataset (n = 6589) after 10‐fold cross‐validation across 3 gene sets. The micro‐average Receiver Operating Characteristic (ROC) curves from the best cross‐validation fold demonstrated stable sensitivity, specificity, and robust discriminative performance in all gene sets. (C) Performance to predict TOO was not different among the 3 optimized ensemble models and corresponding gene sets in the testing dataset (n = 6589). Figure S4: Limit of detection for plasma ctDNA using combined mutation and non‐ mutation features. Clinical samples were serially diluted to different levels of tumor fractions. Sensitivity to detect mutations and non‐mutation gen [file CAM4-14-e71465-s001.docx]

**Table S1. List of clinical samples and sequencing data used in this study**

| **Tissue samples for detection of genomic alterations** | | | |  |  | **N = 272** | |
| --- | --- | --- | --- | --- | --- | --- | --- |
| **DNA-sequencing** | **N = 241** | |  | **mRNA-sequencing** |  | **N = 31** |  |
| Glioma | 2 |  |  | Lung |  | 25 |  |
| Lung cancer | 70 | |  | Breast |  | 3 |  |
| Ovarian cancer | 169 | |  | Colorectal |  | 1 |  |
|  |  |  |  | Glioma |  | 2 |  |
| **Plasma samples for detection of ctDNA** | |  |  |  |  | **N = 55** |  |
| **ctDNA profiling** | **N = 55** | |  | **Treatment, N** |  | **N = 40** |  |
| Stage IV lung cancer | N = 55 | |  | Tyrosine kinase inhibitor |  | 31 |  |
| Age at diagnosis, range (year) | 66 (38 – 84) | | | Immune checkpoint inhibitor |  | 9 |  |
| < 66, N | 23 | |  | **Clinical response*, N (%)** |  | **N = 40** |  |
| ≥ 66, N | 32 | |  | Complete response |  | 1 (2.5) |  |
| Gender, N |  |  |  | Partial response |  | 21 (52.5) | |
| Female | 30 | |  | Stable disease |  | 3 (7.5) |  |
| Male | 25 | |  | Progression disease |  | 15 (37.5) | |
| Histology subtype, N (%) | N = 55 | |  | ** Evaluated by RECIST 1.1 criteria* | |  |  |
| Non-small cell | 55 (100.0) | | |  |  |  |  |
| Adenocarcinoma | 42 (76.4) | | |  |  |  |  |
| Squamous cell carcinoma | 3 (5.5) | |  |  |  |  |  |
| Unknown | 10 (18.1) | | |  |  |  |  |
| Small cell | 0 (0.0) | |  |  |  |  |  |
| **Tissue samples and external data for development of OriCUP model**  **Train and Test sets: (1) TCGA. Validation sets: (2) Robinson *et al** and (3) In-house samples** | | | | | | **N = 10620** | |
| **Cohort** | **(1)** | **(2)** | **(3)** | **Cohort** | **(1)** | **(2)** | **(3)** |
| ACC: Adrenal | 79 |  |  | MESO: Mesothelioma | 87 |  |  |
| BLCA: Bladder | 409 |  |  | OV: Ovarian | 421 | 13 | 8 |
| BRCA: Breast | 1106 | 92 | 106 | PAAD: Pancreatic | 178 | 12 | 7 |
| CESC: Cervical | 304 |  |  | PCPG: Pheochromocytoma | 179 |  |  |
| CHOL: Bile duct | 35 | 25 | 4 | PRAD: Prostate | 501 | 86 | 3 |
| COAD: Colon | 471 | 10 | 63 | READ: Rectal | 166 |  |  |
| DLBC: Lymphoma | 48 |  |  | SARC: Sarcoma | 259 | 57 | 9 |
| ESCA: Esophageal | 184 | 1 | 12 | SKCM: Melanoma | 103 |  |  |
| GBM: Glioblastoma | 157 | 5 | 8 | STAD: Stomach | 412 | 19 | 41 |
| HNSC: Head & neck | 520 |  |  | TGCT: Testicular | 150 |  |  |
| KICH: Kidney chromophobe | 66 |  |  | THCA: Thyroid | 505 |  |  |
| KIRC: Kidney clear cell | 537 | 7 | 7 | THYM: Thymoma | 120 |  |  |
| KIRP: Kidney papillary | 290 |  |  | UCEC: Endometrial | 549 |  | 7 |
| LGG: Low-grade glioma | 516 |  |  | UCS: Uterine | 57 |  |  |
| LIHC: Liver | 371 | 5 | 5 | UVM: Eye melanoma | 80 |  |  |
| LUAD: Lung adernocacinoma | 528 | |  | **Total** | **9889** | **356** | **375** |
|  |  | 24 | 95 |  |  |  |  |
| LUSC: Lung squamous | 501 | |  |  |  |  |  |

* Robinson, D.R., et al., *Integrative clinical genomics of metastatic cancer.* Nature, 2017. **548**(7667): p. 297-303

**Table S2. List of 504 genes**

| *ABL1* | *CDC73* | *ERCC3* | *HIST1H3A* | *LYN* | *NTHL1* | *RAC2* | *SOS1* |
| --- | --- | --- | --- | --- | --- | --- | --- |
| *ACVR1* | *CDH1* | *ERCC4* | *HIST1H3B* | *MALT1* | *NTRK1* | *RAD21* | *SOX17* |
| *AGO2* | *CDK12* | *ERCC5* | *HIST1H3C* | *MAP2K1* | *NTRK2* | *RAD50* | *SOX2* |
| *AKT1* | *CDK4* | *ERF* | *HIST1H3D* | *MAP2K2* | *NTRK3* | *RAD51* | *SOX9* |
| *AKT2* | *CDK6* | *ERG* | *HIST1H3E* | *MAP2K4* | *NUF2* | *RAD51B* | *SPEN* |
| *AKT3* | *CDK8* | *ERRFI1* | *HIST1H3F* | *MAP3K1* | *NUP93* | *RAD51C* | *SPOP* |
| *ALK* | *CDKN1A* | *ESR1* | *HIST1H3G* | *MAP3K13* | *OTX2* | *RAD51D* | *SPRED1* |
| *ALOX12B* | *CDKN1B* | *ETV1* | *HIST1H3H* | *MAP3K14* | *PAK1* | *RAD52* | *SRC* |
| *AMER1* | *CDKN2A* | *ETV6* | *HIST1H3I* | *MAPK1* | *PAK7* | *RAD54L* | *SRSF2* |
| *ANKRD11* | *CDKN2B* | *EZH1* | *HIST1H3J* | *MAPK3* | *PALB2* | *RAF1* | *STAG2* |
| *APC* | *CDKN2C* | *EZH2* | *HIST2H3C* | *MAPKAP1* | *PARK2* | *RARA* | *STAT3* |
| *AR* | *CEBPA* | *EZHIP* | *HIST2H3D* | *MAX* | *PARP1* | *RASA1* | *STAT5A* |
| *ARAF* | *CENPA* | *FAM175A* | *HIST3H3* | *MCL1* | *PAX5* | *RB1* | *STAT5B* |
| *ARID1A* | *CHD7* | *FAM46C* | *HLA-A* | *MDC1* | *PBRM1* | *RBM10* | *STK11* |
| *ARID1B* | *CHEK1* | *FAM58A* | *HLA-B* | *MDM2* | *PDCD1* | *RECQL* | *STK19* |
| *ARID2* | *CHEK2* | *FANCA* | *HNF1A* | *MDM4* | *PDCD1LG2* | *RECQL4* | *STK40* |
| *ARID5B* | *CIC* | *FANCC* | *HOXB13* | *MED12* | *PDGFRA* | *REL* | *SUFU* |
| *ASXL1* | *CREBBP* | *FANCD2* | *HRAS* | *MEF2B* | *PDGFRB* | *RELA* | *SUZ12* |
| *ASXL2* | *CRKL* | *FANCI* | *ICOSLG* | *MEN1* | *PDPK1* | *RET* | *SYK* |
| *ATM* | *CRLF2* | *FANCL* | *ID3* | *MET* | *PFB* | *RFWD2* | *TAP1* |
| *ATR* | *CSDE1* | *FAT1* | *IDH1* | *MGA* | *PGR* | *RHEB* | *TAP2* |
| *ATRX* | *CSF1R* | *FAT4* | *IDH2* | *MITF* | *PHOX2B* | *RHOA* | *TBX3* |
| *AURKA* | *CSF3R* | *FBXW7* | *IFNGR1* | *MLH1* | *PIK3C2G* | *RICTOR* | *TCEB1* |
| *AURKB* | *CTCF* | *FGF19* | *IGF* | *MLH3* | *PIK3C3* | *RIT1* | *TCF3* |
| *AXIN1* | *CTLA4* | *FGF3* | *IGF1* | *MLL2* | *PIK3CA* | *RNF43* | *TCF7L2* |
| *AXIN2* | *CTNNB1* | *FGF4* | *IGF1R* | *MLL3* | *PIK3CB* | *ROS1* | *TEK* |
| *AXL* | *CUL3* | *FGFR1* | *IGF2* | *MPL* | *PIK3CD* | *RPS6KA4* | *TERT* |
| *B2M* | *CXCR4* | *FGFR2* | *IKBKE* | *MRE11A* | *PIK3CG* | *RPS6KB2* | *TET1* |
| *BABAM1* | *CYLD* | *FGFR3* | *IKZF1* | *MSH2* | *PIK3R1* | *RPTOR* | *TET2* |
| *BAP1* | *CYSLTR2* | *FGFR4* | *IL10* | *MSH3* | *PIK3R2* | *RRAGC* | *TGFBR1* |
| *BARD1* | *DAXX* | *FH* | *IL7R* | *MSH6* | *PIK3R3* | *RRAS* | *TGFBR2* |
| *BBC3* | *DCUN1D1* | *FLCN* | *INHA* | *MSI1* | *PIM1* | *RRAS2* | *TMEM127* |
| *BCL10* | *DDR2* | *FLT1* | *INHBA* | *MSI2* | *PLCG2* | *RTEL1* | *TMPRSS2* |
| *BCL2* | *DDX31* | *FLT3* | *INPP4A* | *MST1* | *PLK2* | *RUNX1* | *TNFAIP3* |
| *BCL2L1* | *DICER1* | *FLT4* | *INPP4B* | *MST1R* | *PMAIP1* | *RXRA* | *TNFRSF14* |
| *BCL2L11* | *DIS3* | *FOXA1* | *INPPL1* | *MTOR* | *PMS1* | *RYBP* | *TOP1* |
| *BCL6* | *DNAJB1* | *FOXL2* | *INSR* | *MUTYH* | *PMS2* | *SDHA* | *TP53* |
| *BCOR* | *DNMT1* | *FOXO1* | *IRF4* | *MYB* | *PNRC1* | *SDHAF2* | *TP53BP1* |
| *BIRC3* | *DNMT3A* | *FOXP1* | *IRS1* | *MYBL1* | *POLD1* | *SDHB* | *TP63* |
| *BLM* | *DNMT3B* | *FUB1* | *IRS2* | *MYC* | *POLE* | *SDHC* | *TRAF2* |
| *BMPR1A* | *DOT1L* | *FUBP1* | *JAK1* | *MYCL1* | *PPARG* | *SDHD* | *TRAF7* |
| *BRAF* | *DROSHA* | *FYN* | *JAK2* | *MYCN* | *PPM1D* | *SESN1* | *TRRAP* |
| *BRCA1* | *DUSP4* | *GABRG1* | *JAK3* | *MYD88* | *PPP2R1A* | *SESN2* | *TSC1* |
| *BRCA2* | *E2F3* | *GATA1* | *JUN* | *MYOD1* | *PPP2R2A* | *SESN3* | *TSC2* |
| *BRD4* | *EED* | *GATA2* | *KBTBD4* | *NBN* | *PPP4R2* | *SETD2* | *TSHR* |

**Table S2. (cont.)**

| *BRIP1* | *EGF* | *GATA3* | *KDM5A* | *NCOA3* | *PPP6C* | *SETD8* | *U2AF1* |
| --- | --- | --- | --- | --- | --- | --- | --- |
| *BTK* | *EGFL7* | *GFI1* | *KDM5C* | *NCOR1* | *PRDM1* | *SF3B1* | *UPF1* |
| *CALR* | *EGFR* | *GFI1B* | *KDM6A* | *NCOR2* | *PRDM14* | *SH2B3* | *VEGFA* |
| *CAMTA1* | *EIF1AX* | *GLI1* | *KDR* | *NECTIN4* | *PREX2* | *SH2D1A* | *VHL* |
| *CARD11* | *EIF4A2* | *GLI2* | *KEAP1* | *NEGR1* | *PRKAR1A* | *SHOC2* | *VTCN1* |
| *CARM1* | *EIF4E* | *GNA11* | *KIT* | *NF1* | *PRKCA* | *SHQ1* | *WHSC1* |
| *CASP8* | *ELF3* | *GNAQ* | *KLF4* | *NF2* | *PRKCI* | *SLX4* | *WHSC1L1* |
| *CBFB* | *EP300* | *GNAS* | *KMT2A* | *NFE2L2* | *PRKD1* | *SMAD2* | *WT1* |
| *CBL* | *EPAS1* | *GPS2* | *KMT2B* | *NFKBIA* | *PTCH1* | *SMAD3* | *WWTR1* |
| *CCND1* | *EPCAM* | *GREM1* | *KMT2C* | *NKX2-1* | *PTEN* | *SMAD4* | *XIAP* |
| *CCND2* | *EPHA3* | *GRIN2A* | *KMT2D* | *NKX3-1* | *PTP4A1* | *SMARCA4* | *XPO1* |
| *CCND3* | *EPHA5* | *GSK3B* | *KNSTRN* | *NOTCH1* | *PTPN11* | *SMARCB1* | *XRCC2* |
| *CCNE1* | *EPHA7* | *H3F3A* | *KRAS* | *NOTCH2* | *PTPRD* | *SMARCD1* | *YAP1* |
| *CD274* | *EPHB1* | *H3F3B* | *LATS1* | *NOTCH3* | *PTPRS* | *SMARCE1* | *YES1* |
| *CD276* | *ERBB2* | *H3F3C* | *LATS2* | *NOTCH4* | *PTPRT* | *SMO* | *ZFHX3* |
| *CD79A* | *ERBB3* | *HGF* | *LDB1* | *NPM1* | *PVT1* | *SMYD3* | *ZFTA* |
| *CD79B* | *ERBB4* | *HIST1H1C* | *LMO1* | *NRAS* | *RAB35* | *SNCAIP* | *ZMYM3* |
| *CDC42* | *ERCC2* | *HIST1H2BD* | *LRP1B* | *NSD1* | *RAC1* | *SOCS1* | *ZNF521* |

**Table S3. List of reference samples used in this study**

| **Sample** | **Manufacturer** | **Reference code** | **Validated markers** |
| --- | --- | --- | --- |
| Seraseq® Lung & Brain CNV Mix,  + 3 copies | Seracare (USA) | 0710-0414 | DNA gene amplification |
| *MTAP, CDKN2A* co-loss (CN=0)  reference standard | Cobioer (China) | CBP40132 | DNA gene deletion |
| *MTAP, CDKN2A* co-loss (CN=1)  reference standard | Cobioer (China) | CBP40150 | DNA gene deletion |
| Seraseq® gDNA *BRCA1/2* LGR Inherited Mutation Mix | Seracare (USA) | 0730-0568 | Large genomic rearrangements |
| EMQN01 | EMQN |  | Fusion |
| EMQN02 | EMQN |  | Fusion |
| EMQN03 | EMQN |  | Fusion |
| EMQN04 | EMQN |  | Fusion |
| EMQN05 | EMQN |  | Fusion |
| EMQN06 | EMQN |  | Fusion |
| EMQN07 | EMQN |  | Fusion |
| EMQN08 | EMQN |  | Fusion |
| EMQN09 | EMQN |  | Fusion |
| EMQN10 | EMQN |  | Fusion |


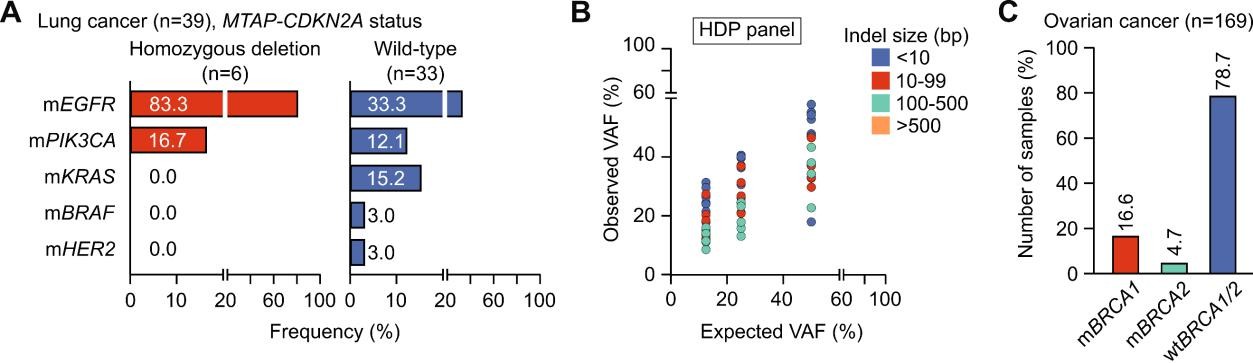


**Figure S1. Performance of FFPE DNA sequencing using high-density probes to determine copy number variation. (A)** Frequency of homozygous *MTAP-CDKN2A* deletion in lung cancer samples having other actionable mutations (n=39). **(B)** *In-silico* simulation of different VAFs for detection of large genomic rearrangement (LGR) in *BRCA1/2* genes. **(C)** Percentage of mutated *BRCA1/2* (m*BRCA*) and wild-type *BRCA1/2* (wt*BRCA*) in ovarian cancer samples (n=169).


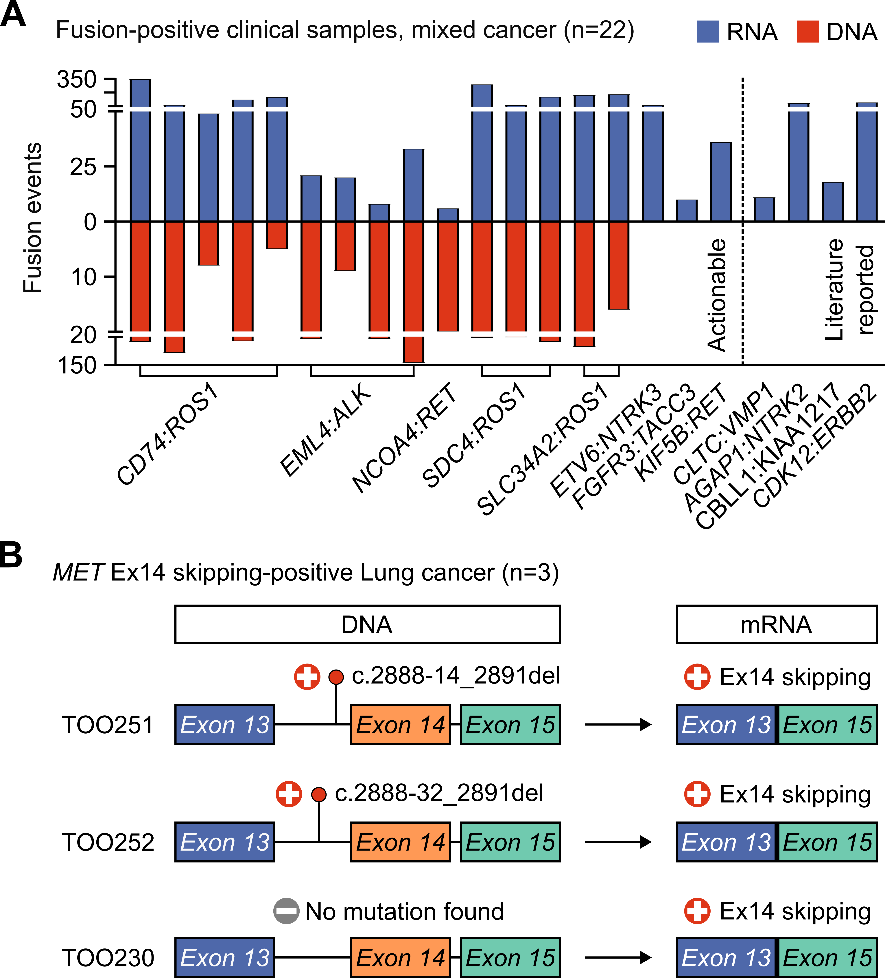


**Figure S2. Performance of FFPE DNA and mRNA sequencing to detect fusion. (A)** In fusion-positive clinical samples, mRNA profiling captured more fusion events and had broader coverage of fusion genes and partners than DNA profiling. **(B)** mRNA profiling could detect MET Ex14 skipping when no DNA mutation was identified in lung cancer samples.


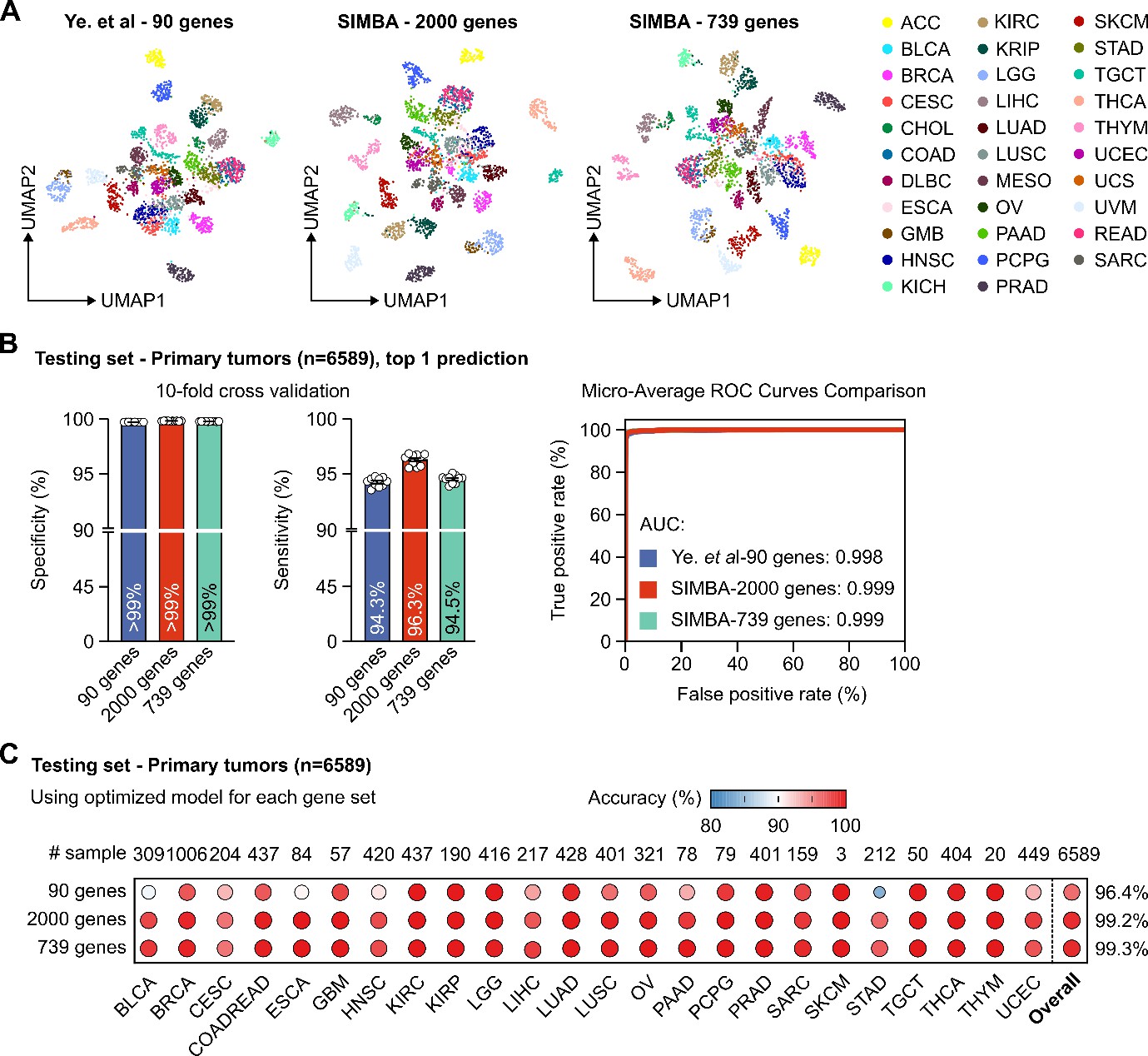


**Figure S3. Performance of mRNA sequencing to predict cancer tissue of origin. (A)** Two- dimensional Uniform Manifold Approximation and Projection for Dimension Reduction (UMAP) using 3 gene sets applied in 32 cancer types in the training dataset revealed distinct clusters corresponding to different cancer types (n=2803). **(B)** Sensitivity and specificity of optimized ensemble models in the testing dataset (n=6589) after 10-fold cross-validation across 3 gene sets. The micro-average Receiver Operating Characteristic (ROC) curves from the best cross-validation fold demonstrated stable sensitivity, specificity, and robust discriminative performance in all gene sets. **(C)** Performance to predict TOO was not different among the 3 optimized ensemble models and corresponding gene sets in the testing dataset (n=6589).


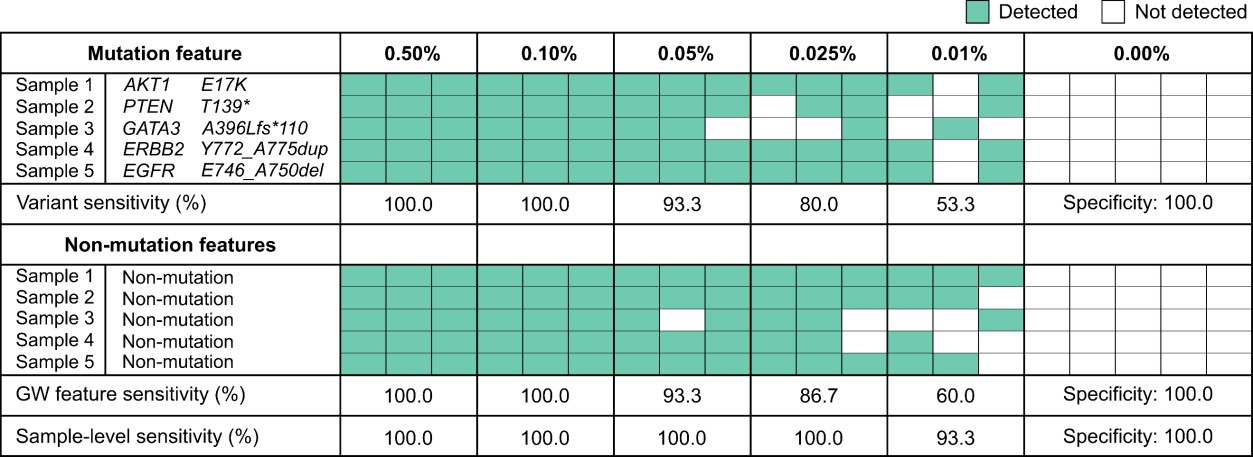


**Figure S4. Limit of detection for plasma ctDNA using combined mutation and non- mutation features**. Clinical samples were serially diluted to different levels of tumor fractions. Sensitivity to detect mutations and non-mutation genome-wide (GW) features were shown. When both mutation and non-mutation features were combined, limit of detection at 90% confidence (LOD90) was determined at 0.01%.
